# Meningeal T cells function in the central nervous system homeostasis and neurodegenerative diseases

**DOI:** 10.3389/fncel.2023.1181071

**Published:** 2023-08-07

**Authors:** Abdellatif Abbaoui, Oluwaseun Fatoba, Toshihide Yamashita

**Affiliations:** ^1^Department of Molecular Neuroscience, Graduate School of Medicine, Osaka University, Osaka, Japan; ^2^World Premier International (WPI)-Immunology Frontier Research Center, Osaka University, Osaka, Japan; ^3^Department of Neuro-Medical Science, Graduate School of Medicine, Osaka University, Osaka, Japan; ^4^Graduate School of Frontier Biosciences, Osaka University, Osaka, Japan

**Keywords:** meningeal T cells, neuroimmunology, meningeal immunity, meningeal lymphatic vessels, homeostasis, behavior, neurodegenerative diseases

## Abstract

Recently, a rising interest is given to neuroimmune communication in physiological and neuropathological conditions. Meningeal immunity is a complex immune environment housing different types of immune cells. Here, we focus on meningeal T cells, possibly the most explored aspect of neuro-immune cell interactions. Emerging data have shown that meningeal T cells play a crucial role in the pathogenesis of several neurodegenerative disorders, including multiple sclerosis, Alzheimer’s, Parkinson’s, and Huntington’s diseases. This review highlights how meningeal T cells may contribute to immune surveillance of the central nervous system (CNS) and regulate neurobehavioral functions through the secretion of cytokines. Overall, this review assesses the recent knowledge of meningeal T cells and their effects on CNS functioning in both health and disease conditions and the underlying mechanisms.

## 1. Anatomy of the meninges

**FIGURE 1:**
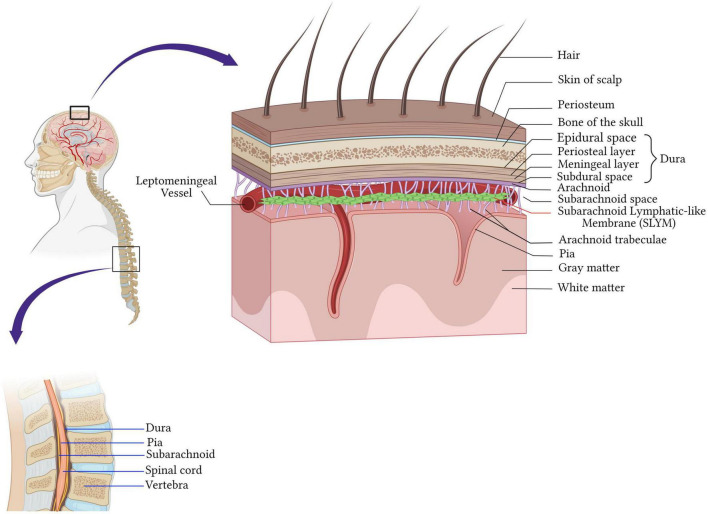
Anatomy of the meninges. The central nervous system is protected by the bone of the skull around the brain and by the vertebrae in the spinal cord. Beneath the bone are the meninges which cover and protect the CNS and consist of four layers membranes; the dura mater (outermost) adjacent to the skull, the arachnoid (intermediate membrane), the pia mater (the most internal, adherent to the brain and spinal cord parenchyma), and the fourth meningeal layer, subarachnoid lymphatic-like membrane (SLYM). SLYM compartmentalizes the subarachnoid space into two functional compartments. Created with BioRender.com.

The central nervous system (CNS) is endowed with well-honed protection. The brain is protected by the cranial cavity, the space within the skull, while the vertebral column protects the spinal cord (Figure 1). The classical scheme is that meninges are three layers of membranes surrounding the brain in the skull, the spinal cord in the spine, and the nerves at the lumbar level. The dura mater (outermost) adjacent to the skull, the arachnoid (intermediate membrane), and the pia mater (the most internal, adherent to the brain and spinal cord parenchyma) constitute the meningeal layers (Figure 1).

Dura mater is a tough fibrous and dense membrane with collagenic and elastic fibers (Protasoni et al., 2011). It is vascularized and well-innervated and contains the lymphatic vessels in rodents and mammalians (Louveau et al., 2015; Absinta et al., 2017). It consists of two layers of dense connective tissue: the outer layer attached to the periosteum and the inner layer, which extends into the vertebral canal, forming the spinal dura mater. The two layers are fused, except in the places where they wrap the sinuses of the dura mater collecting the venous blood of the encephalon directed in the internal jugular veins of the neck (Marieb and Hoehn, 2019; Figure 1).

The arachnoid mater is the middle meningeal layer. It’s a thin layer separating the dura mater and pia mater. The separation of the arachnoid mater from the pia forms a cerebrospinal fluid (CSF)-filled compartment called subarachnoid space. Arachnoid mater epithelial cells have tight junctions, that sit adjacent to the CSF within the subarachnoid space, express drug transport proteins, and serve as physiologic barriers regulating the transport of molecules like the blood-brain barrier (Balin et al., 1986; Yasuda et al., 2013; Figure 1).

Cerebrospinal fluid (CSF) flows through the subarachnoid space located underneath the arachnoid. It’s a rich area of fine filaments of connective tissue called trabeculae that link up the arachnoid to the pia mater (Ghannam and Al Kharazi, 2022), and together are called the leptomeninges.

The deepest layer is the pia mater covering the CNS parenchyma, well vascularized and semipermeable to the CSF in the Virchow-Robin space (Ghannam and Al Kharazi, 2022). Recently, Møllgård et al. (2023) discovered a fourth meningeal layer, known as the subarachnoid lymphatic-like membrane (SLYM). SLYM is an effective barrier for a molecule of more than 3 kilodaltons and compartmentalizes the subarachnoid space into two functional compartments. It is a vascularized layer hosting a considerable population of myeloid cells that proliferate during inflammation and aging (Møllgård et al., 2023).

## 2. Diversity and the origin of meningeal immune cells

**FIGURE 2:**
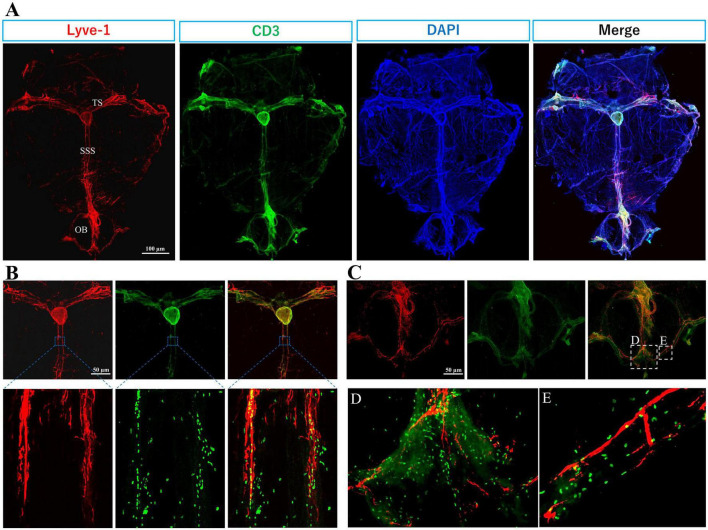
Immune T cells populate the meninges during steady state: The whole mount (dura and arachnoid mater) of 11-week old C57BL/6J mice (*n* = 6) were harvested and stained with anti-CD3 (Cat.No: 555273), anti-Lyve-1 (Cat.No: 67538), and counterstained with DAPI, then imaged using Keyence BZ-X800 microscope, and Olympus FV3000 confocal microscope for **(B)** high magnification. **(A)** T cells (green) distribution is mainly co-localized with meningeal lymphatic vessels (red). Meningeal lymphatics are juxtaposed to sagittal and transverse sinuses. **(B)** High magnification shows T cells within and around superior sagittal sinus (SSS) lymphatics. **(C)** T cell abundance is evident in the olfactory bulb (OB). **(D)** and **(E)** are magnified images from selected areas in merged image of **(C)**. TS, transverse sinus.

**FIGURE 3:**
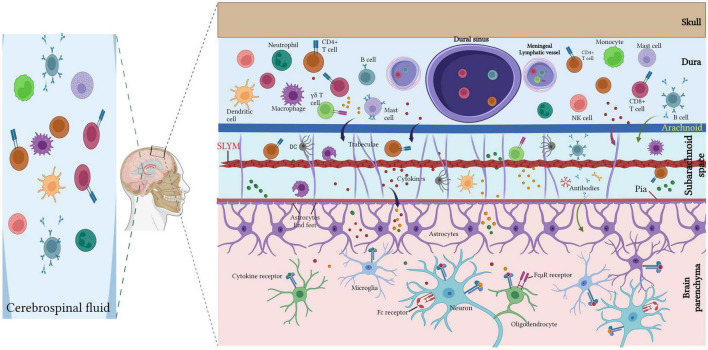
Meningeal immune cells. Diverse immune cell populations (T lymphocytes, B lymphocytes, neutrophils, dendritic cells, macrophages, mast cells) inhabit the meninges under the steady-state with large diversity within the dura mater. Dural immune cells are located nearby the sinuses and the lymphatic vessels. T cell-derived cytokines can directly regulate the neuronal activity via cytokine receptor signaling or indirectly via glial cells (such as microglia and astrocytes) that are able to recognize the cytokines and respond by secreting other cytokines to regulate the neuronal plasticity. The CSF harbor a high amount of central memory T cells, in addition to lesser rate of other immune cell populations of B cells, monocytes, natural killer (NK) cells monocytes and dendritic cells (DCs). Created with BioRender.com.

Diverse immune cells (T lymphocytes, B lymphocytes, neutrophils, dendritic cells, macrophages, mast cells) populate the meninges under healthy conditions and in inflammatory and neurodegenerative diseases conditions (Nayak et al., 2012; Filiano et al., 2017; Korin et al., 2017; Tanabe and Yamashita, 2018; Norris and Kipnis, 2019; van Hove et al., 2019; Ding et al., 2021; Figures 2, 3).

T cells are not present in the CNS parenchyma under steady-state conditions but are largely found within the meninges, especially in the dura mater, and rarely present in the choroid plexus (Ribeiro et al., 2019; Alves de Lima et al., 2020; Figures 2, 3).

The abundance of T cells within the meningeal olfactory bulb (Figure 2C) remains enigmatic. Future investigations will help to unravel their potential role in immune responses in the olfactory bulbs. However, we hypothesize that olfactory bulbs are an exit for T cell and B cells to access the lymphatics at the cribriform plate.

Under steady-state, T lymphocytes (T cells) appear in the meninges starting at birth. Ribeiro et al. (2019) found that a considerable population of γδ T cells accumulate in the meninges space, peak after the first life week (1 week after birth), and slightly decrease with age. Alves de Lima et al. (2020) suggested that γδ T cells inhabit the dural meninges at the perinatal intervals and can be preserved through low-rate self-renewal. Conversely, αβ T cells (CD4 + and CD8 + T cells) start accumulating in the meninges after weaning and are preserved throughout life at a constant level with more CD4+ than CD8+ (Ribeiro et al., 2019). Dural meningeal γδ T cells express interleukin (IL)-17 whereas αβ T cells mainly produce interferon-γ (IFNγ) (Ribeiro et al., 2019; Alves de Lima et al., 2020).

The local bone marrow populations contained in the skull are the main origin of dural immune cells (monocytes and neutrophils) rather than the blood (Cugurra et al., 2021). A recent study using single-cell RNA sequencing, cytometry by time of flight, together with single-cell B cell receptor sequencing showed that dura B cells encompass various developmental stages from pro-B to mature B lymphocytes and that identical B cell subtypes are particularly found in the bone marrow but not in the blood. The selective reconstitution of the skull bone marrow revealed that dura B cells originated from the calvaria (Brioschi et al., 2021; Figure 3). Other recent studies support these data and described the presence of B cells in the dura (Schafflick et al., 2021; Wang et al., 2021). Of interest, parabiosis between wild-type and CD19-Tomato mice demonstrated that B lymphocytes from the systemic circulation rarely infiltrated the meninges under steady-state conditions (Brioschi et al., 2021).

Myeloid cells as well as neutrophils and lymphocytes migrate from the vertebral and calvarial bone marrow to the meninges via specific direct vascular channels crossing the inner skull bone (Herisson et al., 2018; Yao et al., 2018; Cai et al., 2019; Brioschi et al., 2021). Notably, CSF can reach skull bone marrow via dura–skull direct channels and contains proteins that foster the recruitment of various myeloid cells into the meninges (Mazzitelli et al., 2022; Pulous et al., 2022).

Collectively, the meninges are a major hub for diverse immune cells (Figure 3), especially T cells, and act as an important neuroimmune interface for immune surveillance and protection in the meningeal immune niche.

## 3. Chemokines associated with meningeal T cells

Chemokines are a family of small, mostly soluble, proteins that signal through G protein-coupled receptors and are classified into four subclasses (C, CC, CXC, and CX3C) (Raz and Mahabaleshwar, 2009). Chemokines are chemotactic cytokines that regulate migration patterns and positioning of immune cells and promote proliferation and cell survival. Their most studied function is the attraction (chemotaxis) and the control of the activation state of the immune system cells. Different chemokine signalings were involved in T cells migration into the CNS during autoimmune disease and meningeal inflammation [Reviewed in Täuber and Moser (1999) and Heng et al. (2022)].

Other functions have also been attributed to chemokines during embryonic development, physiology, and pathology of the nervous system (Raz and Mahabaleshwar, 2009; Ramesh et al., 2013; Wang and Knaut, 2014).

Different chemokines and their receptors are associated with T cell functions. Single nucleus RNA-sequence data showed that meningeal T cells express different chemokine receptors involved in trafficking and T cell’s function specifically CXCR6, CXCR3, CXCR4, CCR2, CCR7, and CCR5 (van Hove et al., 2019).

In parallel, it appears also that dural-resident myeloid cells express all the associated chemokine ligands: CXCL16, CXCL12, CXCL9, CXCL10, CCL2, CCL12, CCL3, and CCL5 (van Hove et al., 2019). This suggests that locally secreted chemokines play a key role in meningeal T cell recruitment, migration, and function.

CXCR6 is highly expressed by dura meningeal γδ17 T cells and the CXCL16-CXCR6 pathway is involved in γδ17 T cells recruitment, maintenance, and/or activation in the dura mater (Alves de Lima et al., 2020). Moreover, δ TCR-positive cells express chemokine retinoic acid-related orphan receptor-γt (RORγt) and C-C motif chemokine receptor 6 (CCR6) (Ribeiro et al., 2019; Alves de Lima et al., 2020), a chemokine receptor implicated in the migration of γδ17 T cells to the dermis. In addition, CCR2 drives rapid γδ17 T cell recruitment to inflamed tissues during autoimmunity, cancer, and infection (McKenzie et al., 2017). The CCL21-CCR7 pathway is another crucial axis for T cells and dendritic cells (DCs) to circulate across the lymphatic system, and it facilitates cells migration in physiological and pathological conditions (Debes et al., 2005; Weber et al., 2013). DC movements in the mouse’s skin are guided by endogenous gradients of the chemokine CCL21 to migrate directionally toward CCL21-expressing lymphatic vessels (Weber et al., 2013). Louveau et al. (2018) found that meningeal T cells migration into the cervical lymph nodes is CCR7-dependent and that CCR7+ T cells are principally in close relationship with CCL21+ lymphatic endothelial cells. Similarly, CCR7-KO mice have a high number of meningeal T cells and partial exclusion of the meningeal T cells from the lymphatic compartment (Louveau et al., 2018). In addition, reduced expression of CCR7 by meningeal T cells in aged mice resulted in increased effector and regulatory T cells in the meninges and decreased T cells retention in the deep cervical lymph nodes (dCLNs) (da Mesquita et al., 2021). CCR5/CXCR3 Chemokine signaling pathway promotes T cells adhesion to the leptomeninges and their migration from the CSF into the CNS parenchyma (Schläger et al., 2016).

Taken together, these findings suggest diverse chemokines signaling regulate T cell migration, adhesion, and activation in the meninges.

## 4. Meningeal T cells influence central nervous system homeostasis

**FIGURE 4:**
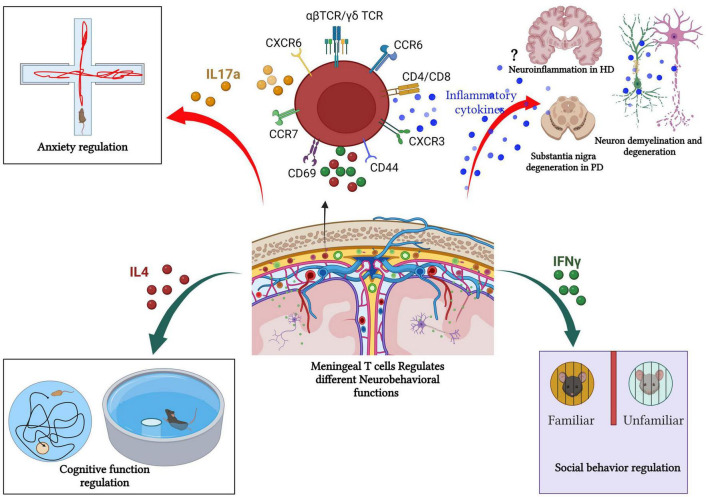
Meningeal T cells in health and diseases. Meningeal T cells regulate several neurobehavioral functions (such as cognitive function, social interaction, and anxiety like-behavior) in steady state. However, during pathologic processes, such as neurodegeneration, meningeal T cells promote disease symptomatology and progression by augmenting the production of proinflammatory mediators. In PD and MS, the role of T-cell in fostering neurodegeneration and cognitive decline is well documented. In other neurodegenerative diseases such as HD, the link between meningeal T cells, neuroinflammation, and disease progression remains unclear as T cell frequency is unchanged in human HD postmortem brain compared with healthy patient brain tissue. Created with BioRender.com.

Accumulating evidence highlights the key role of T cells in CNS homeostasis. The story started when Kipnis et al. (2004) found that mature T cell deficiency disturbs the performance of mice in cognitive tasks. Severe combined immune deficiency (SCID) and nude mice (deficient in mature T cells) showed restored cognitive functions and increased hippocampal neurogenesis following T cell repopulation (Kipnis et al., 2004; Brynskikh et al., 2008; Wolf et al., 2009). However, B cell-deficient mice do not show any changes compared to their wild-type littermates in the cognitive tasks. Consequently, the adoptive transfer of CD4+ T cells, but not CD8+ T cells led to improved spatial learning and memory performances, decreased hippocampal neurogenesis, and reverted anxiety-like behavior (Rattazzi et al., 2013; Radjavi et al., 2014; Figure 4).

Interleukin-4 (IL-4) is a pleiotropic cytokine secreted mostly by T helper 2 (Th2) lymphocytes and is known to drive CD4+ T cell polarization in the Th2 phenotype. IL-4 physiological functions are mediated by the IL-4 receptor (IL-4R) complex (Silva-Filho et al., 2014). Of interest, wild-type (WT) mice subjected to cognitive tasks showed increased T cell-derived IL-4 in the meningeal space (Derecki et al., 2010). Furthermore, IL-4-deficient mice displayed a polarized proinflammatory meningeal myeloid cell phenotype and cognitive deficits. To understand how meningeal T cells support cognitive function, Derecki et al. (2010) depleted T cells from the meningeal space in WT mice using an anti-VLA4 antibody, a potent inhibitor of T cell migration. Acute depletion of meningeal T cells in WT mice led to impaired memory performance and polarized meningeal myeloid cells toward proinflammatory phenotypes (Derecki et al., 2010). Conversely, passive T cells transfer from WT mice into IL-4 deficient mice restored cognitive impairment and reduced the proinflammatory phenotype of meningeal myeloid cells. Brain-derived neurotrophic factor (BDNF) is known to play an important role in cognitive tasks (Hall et al., 2000; Choy et al., 2008). Remarkably, while WT mice exhibited a considerable increase in BDNF mRNA levels after cognitive task performance, IL-4-deficient mice showed no increase (Derecki et al., 2010). Similarly, primary astrocytes treated with IL-4 *in vitro* demonstrated increased BDNF mRNA levels, implicating IL-4 as an important factor for the maintenance of BDNF production after the cognitive task.

Wolf et al. (2009) have also revealed a significant decrease in BDNF production and hippocampal neurogenesis following CD4+ T cells depletion. However, other evidence has shown that IL-4 inhibits hippocampal adult rat neural stem cell proliferation *in vitro*, and regardless of high levels of IL-4 in the CNS, mice with restricted CD4+ T cell receptor repertoire showed an impaired cognitive performance and hippocampal neurogenesis (Jeon et al., 2016). Therefore, more investigations are needed to clarify the potential role of CD4+ T cells-derived IL-4 in cognitive function control. CD4+ T cells are not present in the adult hippocampus and CNS parenchyma, raising the question of where these cells release their cytokine IL-4. Perhaps, they are typically present in the dura mater, and CSF serves as the main route driving meningeal T cells cytokines to the neural cells within different CNS regions through paravascular influx toward glial limitans (Roth et al., 2014). Additionally, CNS antigens might be the potential key molecules instructing release activity of cytokines by different meningeal T cell subtypes.

Together, these findings suggest the potential role of T cells-derived IL-4 in the control of cognitive function through meningeal myeloid cell phenotype and astrocytic BDNF release.

T cell and their cytokines regulate other behavioral functions including social behavior (Filiano et al., 2016), anxiety and obsessive-compulsive-like behaviors (Rattazzi et al., 2013; Alves de Lima et al., 2020), posttraumatic stress disorder (Cohen et al., 2006), juvenile social and maternal behavior (Quinnies et al., 2015).

Filiano and colleagues demonstrated that SCID mice (T and B cell-deficient mice) showed impaired social behavior as evidenced by the three-chamber sociability assay. Unlike WT mice, SCID mice fail to exhibit a preference interaction with another mouse over exploring an inanimate object (Filiano et al., 2016). However, repopulating SCID mice with WT-derived T cells reversed the impaired social preference. Furthermore, increased T cells level within the healthy meninges promoted IFNγ production and restored social preference in IFNγ deficient mice (Figure 4). Also, conditional deletion of IFNγ receptors from prefrontal cortex GABAergic inhibitory neurons induced a noticeable social behavioral deficit similar to the SCID mice (Filiano et al., 2016). Conversely, intracerebroventricular (ICV) injection of recombinant IFNγ in SCID mice improved the impaired social behavior (Filiano et al., 2016). These findings suggest that meningeal T cells-mediated cytokine release contributes to neuronal circuit functions and related behaviors.

Meningeal γδ T cells producing IL-17a play a vital role in the control of anxiety-like behavior (Figure 4). γδ T cells are present in the dural meninges since birth with a high proliferation rate at early postnatal age, then continue with age by slow self-renewal for life and are preserved as tissue-resident cells (Alves de Lima et al., 2020) suggesting an important neurophysiological role of these special T cells subtype given.

γδ T (Tcrd−/−) deficient mice showed an obvious anxiety-like behavior as revealed by elevated plus maze and open field tests, but no alteration of cognitive function or social preference. Specific depletion of meningeal γδ17 T cells by intra-cisterna magna injection of anti-TCR γδ antibodies significantly decreased anxiety behavior. Additionally, anti-TCR γδ treatment reduced meningeal IL-17a-producing cells. Il-17a-/- mice or injection of anti-IL-17a neutralizing antibodies into the cisterna magna in WT mice showed a significant decrease in anxiety-like behavior. However, delivery of recombinant IL-17a into the CSF of Tcrd−/− mice led to increased anxiety behavior, indicating the role of γδ T cells expressing Il-17a as potent homoeostatic regulators of anxiety (Alves de Lima et al., 2020). The medial prefrontal cortex (mPFC) is known to be an essential brain area for danger interpretations and regulating cortical responses (Calhoon and Tye, 2015).

IL-17Ra is expressed by mPFC glutamatergic neurons under a steady state. Remarkably, conditional depletion of mPFC IL-17Ra alleviates anxiety in mice, further implicating the meningeal γδ17 T as regulator of anxiety-like behavior in mice via neuronal IL-17Ra signaling (Alves de Lima et al., 2020).

Other studies have implicated IL-17a in mouse behavior. Of interest, in maternal immune activation (MIA)-induced neurodevelopmental conditions, such as autism spectrum disorder (ASD), maternal IL-17-a induces abnormal cortical development and behavioral abnormalities in offspring, potentially by IL-17Ra receptor signaling in neurons (Choi et al., 2016). However, in both adult MIA offspring and monogenic mutant mice, IL17-a injection into the primary somatosensory cortex dysgranular zone (S1DZ) promotes the sociability behavior via IL-17Ra signaling in S1DZ neurons indicating a complex mechanism underlying IL-17a regulation of behavior in mice (Reed et al., 2020).

These findings foster our understanding concerning molecular mechanisms regulating neural–immune communications and indicate an evolutionary association between mood, social behaviors, and cytokines (such as IL-4, IL-17a, and IFN-γ) signaling.

## 5. Meningeal T cells behavior during neuroinflammation and neurodegenerative diseases

Many studies have demonstrated the involvement and importance of T cells and meningeal immunity during neuroinflammatory processes, including traumatic brain injury and strokes. This review focuses on the functional and pathogenic roles of meningeal T cells in neurodegenerative diseases such as multiple sclerosis, Parkinson’s disease, Huntington’s disease, and Alzheimer’s disease.

### 5.1. Multiple sclerosis

Multiple sclerosis (MS) is a chronic autoimmune inflammatory disease that affects the CNS (the brain and spinal cord). It begins with immune system dysfunction and immune cell infiltration into the CNS causing neuronal demyelination, deficits in motor, sensory, and cognitive functions, as well as visual disturbances that can progress to an irreversible handicap (Compston and Coles, 2008; Dendrou et al., 2015). Leakage of the blood–brain barrier and T cells, especially CD8+ T cells, are observed early in MS lesions (Dendrou et al., 2015). Infiltrated T cells are found within the CNS parenchyma, the meninges, and the CSF (Giunti et al., 2003; Alvermann et al., 2014). Previous study showed that only meningeal-activated T cells are authorized to access the CNS parenchyma in Lewis rat experimental autoimmune encephalomyelitis (EAE), a model of multiple sclerosis (Schläger et al., 2016; Figure 4).

In MS mouse model and human patients with MS, ectopic lymphoid follicles are present in the meninges. These organized aggregates of immune cells and antigens constitute a favorable environment for T cell reactivation and correlate with disease severity and inflammation (Russi and Brown, 2015). Recent evidence revealed that dura mater and its lymphatics marginally contribute to EAE. The leptomeninges showed a strong inflammation and structural changes with a massive infiltration of T cells and myeloid cells, while the dura mater was slightly affected. Additionally, in rat and mouse EAE models, T cells are more strongly activated in the leptomeninges than in the dura (Merlini et al., 2022).

In the adoptive transfer of autoreactive T cells (passive EAE), T cells appear in the dura mater (Kwong et al., 2017) and the leptomeninges before the onset of clinical symptoms, indicating that the leptomeninges represents a strong checkpoint for T cells infiltration into the CNS parenchyma during autoimmune inflammation (Bartholomäus et al., 2009; Schläger et al., 2016). Using active EAE by immunization, Louveau et al. (2018) demonstrated that infiltrated T cells appear in the meninges before appearing in the parenchyma and before clinical symptoms development.

Bartholomäus et al. by using Intravital two-photon imaging of myelin basic protein (MBP)-specific GFP-labeled T cells (TMBP-GFP cells) in the lumbar spinal cord during the passive EAE, found that the first arriving T cells appear in the subarachnoid area 1 day after transfer, and stayed close to pial blood vessels, crawling on surfaces in the outline of the vessels in a very late antigen-4 (VLA-4)-dependent manner, but not lymphocyte function-associated antigen-1 (LFA-1) dependent manner (Bartholomäus et al., 2009). Diverse interactions with meningeal/paravascular macrophages and cognate antigen recognition are required for activation of autoreactive T cells that respond by upregulating proinflammatory cytokines [IFN-c, IL-17, tumor necrosis factor (TNF)-a, IL-2], proteases, chemokines and chemokine receptors (CCL5, CCR5, CXCR3, CXCR4) (Bartholomäus et al., 2009). Also, chemokines released by CNS-associated macrophages are necessary for T cell recruitment and transmigration into the leptomeninges. Moreover, contact with resident antigen-presenting cells (APCs) (meningeal, perivascular, and choroid-plexus macrophages, and parenchymal microglia) induced activation of autoreactive T cells, suggesting that meningeal reactivation of T cells is needed for parenchymal raid of immune infiltrates prompting CNS damage (Lodygin et al., 2013; Rua and McGavern, 2018).

In human patients with MS, granulocyte–macrophage colony-stimulating factor (GM-CSF) and CXCR4 define a T-helper cells signature in MS, emphasizing CXCR4 and GM-CSF signaling as a potent mechanism stimulating T cell transmigration into the CNS (Galli et al., 2019). Concomitantly, resident macrophages and dendritic cells (DCs) express the chemokines (such as CXCL12), necessary for parenchymal invasion, tissue damage, and disease severity (Ransohoff et al., 2003; Greter et al., 2005).

In rat model of EAE, the lymph nodes have been demonstrated to serve as an essential site of autoimmune T cell licensing and where profound functional change takes place before CNS infiltration (Flügel et al., 2001). Another piece of evidence showed that transferred T cells acquire a specific encephalitogenic phenotype needed for CNS entrance after a transitory stay in the lungs, a niche for powerful autoaggressive T cells (activated T cell and myelin-reactive memory T cells) responsible for autoimmune disease (Odoardi et al., 2012).

Recent evidence highlighted the potential role of meningeal lymphatic vessels (MLV) as a route for CSF immune cells drainage into the deep and superficial cervical lymph nodes (dCLNs and sCLNs), and that impairment of MLV function promotes disease progression in neurodegenerative disorders (Ding et al., 2021). However, the ablation of MLV reduced the EAE pathology progression and inflammation response of brain-reactive T cells (Louveau et al., 2017). Similarly, ablation of lymphatics of the nasal side of the cribriform plate does not show any substantial changes, supporting the role of MLV as immune surveillance of the CNS and the main route driving the CNS-infiltrating T cells drain into CLNs (Louveau et al., 2018).

In the absence of meningeal lymphatic drainage, RNA-sequencing analysis of myelin oligodendrocyte glycoprotein (MOG)-specific T cells in the dCLNs showed a profound genetic change preventing the acquisition of the encephalitogenic profile. This emphasizes the potential role of lymphatic drainage in regulating meningeal T cell phenotype in response to CNS antigens (Louveau et al., 2018).

### 5.2. Parkinson’s disease

Parkinson’s disease (PD), the second most common neurodegenerative disease, is an age-dependent neurodegenerative and neuroinflammatory disease, characterized by progressive loss of dopaminergic neurons in the substantia nigra and the striatum, together with the alpha-synuclein aggregation and Lewy body inclusions (Dickson, 2012).

Numerous studies have demonstrated T cell involvement in the pathophysiology of PD. T cells infiltrate into the brain in different animal models of PD, including 6-hydroxydopamine (6-OHDA)-induced neurodegeneration in mice and rats (Theodore and Maragos, 2015; Ambrosi et al., 2017), the 1-methyl-4-phenyl-1,2,3,6-tetrahydropyridine (MPTP) model in mouse (Reynolds et al., 2010; González et al., 2013), human α-synuclein (α-syn)-overexpressing transgenic mice (hαSyn, Thy1-SNCA) (Sommer et al., 2016; Iba et al., 2020). Other studies demonstrated that T cell-deficient mice were resistant to MPTP-induced neurodegeneration (Benner et al., 2008; Brochard et al., 2009; González et al., 2013). By contrast, T cell-deficient rats injected with AAV9-α-syn showed no significant changes in microglial activation and neuronal loss (Subbarayan et al., 2020), indicating a pivotal role of T cell reaction during the pathological processes in PD (Figure 4).

Few studies have investigated the dynamic and meningeal T cell activity during PD development and meningeal lymphatics functionality. An interesting study conducted by Zou et al. (2019) showed that blocking meningeal lymphatic drainage in A53T mice, overexpressing mutated form of humanα-synuclein, aggravated glymphatic dysfunction leading to severe Parkinson’s disease-like pathology including severe accumulation of α-syn, glial activation, inflammation, dopaminergic neuronal loss, and motor deficits. Remarkably, a significant increase in CD3+, CD4+, and CD8+ T cell numbers accumulated in the meninges and decreased within the deep cervical lymph nodes (Zou et al., 2019). A recent study by Ding et al. (2021) also revealed that mice injected withα-syn preformed fibrils present pathological outcomes, delayed meningeal lymphatic drainage, loss of tight junctions among meningeal lymphatic endothelial cells, and enhanced meningeal inflammation. The ligation of the mLV induced worse pathological and behavioral outcomes (Ding et al., 2021).

Currently, no studies have specifically explored how meningeal immune T cells contribute to PD pathogenesis, as such, further investigations are needed to clarify the dynamics and effects of meningeal T cells during the disease progression in PD and underlying molecular mechanisms.

### 5.3. Huntington’s disease

Huntington’s disease (HD) is a fatal, autosomal dominant inherited neurodegenerative disorder caused by abnormal polyglutamine repeat expansion in the exon 1 of the huntingtin gene placed on chromosome 4 (The Huntington’s Disease Collaborative Research Group, 1993). It is known that the pathogenesis of HD is associated with neuroinflammation, but remains unclear how the immune system affects the disease progression in HD. Several studies have demonstrated that increased pro-inflammatory cytokines (such as plasma IL-6 and IL-8 IL-4, IL-10, and TNF-α levels) correlate significantly with disease progression and severity in HD (Björkqvist et al., 2008; Rocha et al., 2016; Figure 4).

The premanifest stage of HD is characterized by an increased prevalence of IL-17–producing T helper 17.1 (Th17.1) cells in the CSF of HTT gene expansion carriers compared with the healthy controls, indicating a positive correlation between CSF Th17.1 frequency and HD symptomatology and disease progression (von Essen et al., 2020). Unlike other neurodegenerative diseases, such as PD and amyotrophic lateral sclerosis, where T cells activity (CD4+ T and NKT cells) is evident (Rentzos et al., 2012), T cell frequency is unchanged in human HD postmortem brain compared with healthy patient brain tissue (Silvestroni et al., 2009). Park et al. (2019) showed reduced levels of iNKT cells at the disease onset in R6/2 HD transgenic mice. However, iNKT deficiency does not affect HD progression in R6/2 transgenic mice. Interestingly, repeated activation of iNKT cells with α-GalCer increased iNKT cells infiltration into the brain and accelerated HD progression in R6/2 Tg mice, suggesting a potential link between iNKT cells and HD disease progression (Park et al., 2019). The origin of infiltrated iNKT cells was not investigated in this study and it will be interesting to look at meningeal immune T cells and the dynamic of different subsets during HD disease development.

### 5.4. Alzheimer’s disease

Alzheimer’s disease (AD) is the most prevalent neurodegenerative disease in the elderly and the most common cause of dementia (Scheltens et al., 2021). Age is the main risk factor for late-onset AD, and extracellular deposit of senile plaques composed of amyloid beta (Aβ) and tau protein hyperphosphorylation mediated intracellular neurofibrillary tangles are its fundamental neuropathological hallmarks (Hyman et al., 2012).

Studies in human patients and mouse models highlighted the potential role and dysfunction of peripheral T cells in AD (Dai and Shen, 2021; Guo et al., 2023). Recent studies have shown an increased infiltration of T cells into the brain of old mice and in the plaque-harboring cortex of AD-prone mice (Mrdjen et al., 2018). Gate et al. (2020) have shown increased CD3+CD8 T cells within the hippocampus of patients with AD compared with the healthy brain and detected close to Aβ plaques and neuronal structures. Notably, CD3+CD8+ T cells were also detected in the leptomeninges adjacent to the hippocampus of patients with AD (Gate et al., 2020). More so, the CSF investigation showed that clonally expanded CD8+ T effector memory CD45RA+ (TEMRA) cells, with high expression of cytotoxic effector genes including natural killer cell granule protein 7 and granzymes A, patrol the CSF in the brains with AD (Gate et al., 2020). Additionally, transcriptomic profiling of CD8+ T cells isolated from amyloid precursor protein-presenilin-1 (APP-PS1) double-transgenic mouse model of AD and aged WT mice brain identified them as tissue-resident memory (Trm) T cells with upregulated genes involved in type I IFN signaling [Ifnar2, Irf9, and several IFN-stimulated genes (ISGs)]. However, cytotoxic granzyme M gene was downregulated and granzyme B expression was not detected (Altendorfer et al., 2022), suggesting that brain CD8+ T cells promote neurodegeneration via ISGs expression.

Aging leads to progressive impairment of meningeal lymphatics and CSF drainage (Ma et al., 2017; Ahn et al., 2019). Ablation of meningeal lymphatics in young-adult 5× familial Alzheimer’s disease (5× FAD) mice promotes amyloid-β accumulation in the meninges and brain (Da Mesquita et al., 2018). Furthermore, treatment of aged mice with vascular endothelial growth factor C improves meningeal lymphatic drainage of macromolecules from the CSF, leading to cognitive function improvement (Da Mesquita et al., 2018). This finding indicates that meningeal lymphatics dysfunction is a potential disease-aggravating factor in age-related cognitive decline and neurodegeneration and may impair the procognitive functions of meningeal T cells.

It remains unclear if the infiltrated and circulating T cells in the AD brains and CSF, respectively, originated from the meninges. Further investigations are warranted to better understand the dynamic of meningeal T cells during disease progression in AD. Immunophenotyping and transcriptomic profiling will improve our understanding of the role and the potential effects of different meningeal T cell subtypes in neurodegeneration process during AD progression.

## 6. Conclusion

Meninges are complex structural tissue harboring a wide population of immune cells. Meningeal immune cells have a potential effect on neurons and CNS functions together with related behaviors like anxiety, social behavior, spatial learning, and memory. Their location in the meninges around the dural venous sinuses and blood vessels is adapted to assure continued surveillance and response to any pathogen that may threaten the CNS parenchyma. Also, meningeal immune cells regulate sustainable monitoring of CNS by inspection of different antigens released and circulating within the CSF. It is also clear that meningeal immune cells affect behavior, especially T cells, which differ depending on the microenvironment conditions and could be very harmful during CNS neuroinflammation.

Despite the extensive findings supporting the diversity and roles of meningeal immune cells, especially T cells, we are just beginning to understand the origins, development, and functions of these cell populations, and many questions are yet to be clarified. Some of the intriguing questions include: are outside structures of the meninges, specifically the skull and the bone marrow the only origin of immune cells populating the meninges? Could CNS parenchyma be another source? Do meningeal immune cells create a tissue-resident population through local intrameningeal proliferation and developing autonomous life from the peripheral immune system, or they are renewed by continued infiltration from outer layers? What mechanisms govern the infiltration of meningeal T cells into the CNS in health and disease? What are the roles of meningeal lymphatics in regulating this process? Do immune T cells modify the tight junction structure of the meninges to migrate into the CNS? If so, what mechanism(s) is/are involved? Do the meningeal layers contain some lacunae that allow T cells and other immune cells to infiltrate into the CNS? In addition, the kinetics and phenotype of meningeal immune T cell subpopulations responding to disease progression in neurodegenerative diseases are not fully understood. A deep understanding of cellular and molecular mechanisms involved in different interactions between meningeal immune cells and CNS parenchyma could foster our understanding of the etiology of CNS disorders and promotes the identification of promising therapeutic targets for autoimmune and neurodegenerative diseases.

## Author contributions

AA searched the literature and drafted the manuscript. OF and TY critically revised the manuscript. All authors have made a substantial, direct, and intellectual contribution to the work, and approved it for publication.
